# Supplementary material for: Olfactory system structure and function in newly hatched and adult locusts
Source: Sci Rep. 2024 Jan 31;14:2608. doi: 10.1038/s41598-024-52879-7 (PMC10830560; doi:10.1038/s41598-024-52879-7)

Olfactory system structure and function in newly hatched and adult locusts

by

Kui Sun[1*], Subhasis Ray[1,2*], Nitin Gupta[1,3], Zane Aldworth[1], and Mark Stopfer[1§]

[*] co-equal contributions, [§] for correspondence

[1] Eunice Kennedy Shriver National Institute of Child Health and Human Development, National Institutes of Health, Bethesda, MD, USA

[2] current address: Plaksha University, Sahibzada Ajit Singh Nagar, Punjab, India

[3] current address: Indian Institute of Technology Kanpur, Kanpur 208016, India

Supplementary Figure 1

Left: LFP response when the antenna of an adult locust is cut after segments 7 and then after segment 10. Right: Spectrogram of the LFP response shows decreasing oscillation frequency as antennal segments are removed. Black horizontal bar: 1 sec odor pulse.

Supplementary Figure 1

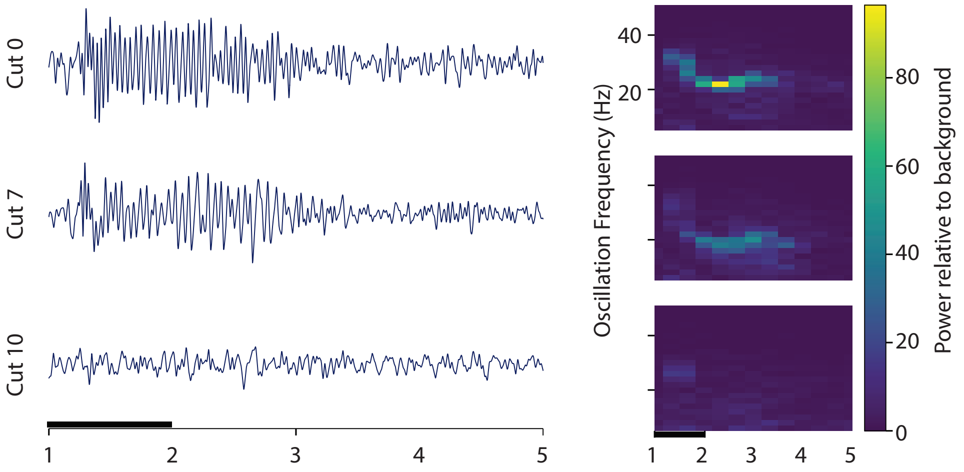

Supplement: Supplementary file 1 — Supplementary Figure 1. [file 41598_2024_52879_MOESM1_ESM.pdf]
